# Synthesis of NiO nanostructures as electrode materials for the voltammetric determination of epinephrine in the presence of acetaminophen

**DOI:** 10.5599/admet.3201

**Published:** 2026-05-09

**Authors:** Mais A. Mohammed, Shemaa A. Soud, Reem Adham Al-Bayati, Shimaa B. Al-Baghdadi

**Affiliations:** 1 Department of Applied Chemistry, College of Applied Sciences, University of Technology- Iraq; 2 Department of Biotechnology, College of Applied Sciences, University of Technology, Baghdad, Iraq; 3 Chemistry Department, Al-Mustansiriyah Unersity, Iraq; 4 Energy and Renewable Energies Technology Research Center, University of Technology, Baghdad, Iraq

**Keywords:** Voltammetric measurement, screen-printed electrode, disposable sensor, real sample analysis

## Abstract

**Background and purpose:**

Epinephrine, also called noradrenaline, is an important chemical mediator in the central nervous system of mammals.

**Experimental approach:**

The hydrothermally synthesized NiO nanostructures were used for the modification of a screen-printed electrode, and they were characterized and used in this work for the voltammetric determination of epinephrine in the presence of acetaminophen. Several electrochemical techniques were employed to investigate the electrochemical properties of NiO-modified screen-printed carbon electrode, including cyclic voltammetry, differential pulse voltammetry and chronoamperometry.

**Key results:**

The differential pulse voltammetry peak current of epinephrine was linear with concentration in the range from 0.01 to 400.0 μmol L^-1^, and the limit of detection was 0.005 μM with a sensitivity of 0.1167 μA L μmol^-1^. The findings indicated that the current signals for epinephrine were significantly amplified, a result attributed to the superior catalytic performance of the NiO nanostructures. Furthermore, the oxidation peaks for epinephrine and acetaminophen were distinctly separated, with potential differences of approximately 360 mV and 545 mV, respectively.

**Conclusion:**

Furthermore, the NiO modified screen-printed carbon electrode was successfully applied to quantify epinephrine and acetaminophen in both urine samples and pharmaceutical formulations. The results indicated satisfactory recovery rates for the target analytes. Consequently, this electrode is suitable for the analysis of both compounds in pharmaceutical and clinical laboratory settings.

## Introduction

Epinephrine (EP), also known as noradrenaline, is an important chemical mediator in the central nervous systems of mammals. EP occurs in nervous tissues and biological fluids, mainly as an organic cation [1,2]. From a chemical standpoint, EP belongs to the catecholamine family. This hormone is produced only in the adrenal glands and is derived from the amino acids phenylalanine and tyrosine. EP is a highly influential factor in biological functions and neurochemical activities within the human body. It increases heart rate, causes vasoconstriction, and increases the blood supply to muscle tissue. It can be employed to deal with different types of medical emergencies, including a major allergic shock (anaphylaxis), the stoppage of heart activity (cardiac arrest) or a slight external haemorrhage [3,4]. Repeated injections of EP may cause serious adverse reactions such as haemorrhaging in the brain due to the sudden increase in arterial pressure and disturbances in heart rhythm. However, there are more serious adverse effects associated with repeated injections of EP, such as intracranial bleeding owing to the sudden increase in arterial pressure and disturbances in heart rhythm [5]. Thus, the determination of EP levels is particularly significant in studying neural functions and other biological processes.

Acetaminophen (AC), N-acetyl-para-aminophenol, or paracetamol, is a well-established drug used extensively around the world. This common medication works as an antipyretic for the alleviation of fever and also as an analgesic agent for pain reduction, therefore being used worldwide as a potent medication to relieve mild to moderate pains related to headaches, arthritis and post-operations [6,7]. Moreover, it has no carcinogenic potential and, when a patient is intolerant to aspirin, it is a good alternative, since at recommended doses its safety record is well established. Acetaminophen is predominantly metabolized through the liver, so an overdose can result in the production of highly toxic intermediates to the point where, without proper treatment, serious liver damage can occur [8,9].

In addition, the use of AC is associated with increased brain serotonin (5-HT) levels. The elevation in serotonin levels can then lead to inhibition of the liver enzyme tryptophan-2,3-dioxygenase (TDO). It is also well established that 5-HT can markedly influence epinephrine secretion in the brain. Conversely, the mechanism of action of AC primarily involves reducing the synthesis of cyclooxygenase-derived compounds in the central nervous system. The activating influence of AC on cyclooxygenase function in viable cells can be counteracted by the presence of EP [10,11]. Therefore, developing a straightforward, economical, and highly responsive method to detect both EP and AC at the same time would be highly advantageous.

Various analytical techniques, including flow injection analysis [12,13], chromatographic methods [14,15], and ultraviolet-visible (UV-Vis) spectrophotometry [16,17], have been utilized for the concurrent detection of EP and AC. Nonetheless, the widespread implementation of those strategies has been hindered by their operational complexity, prolonged periods and significant price.

Because EP and AC have electroactivity, they can be quantified with electrochemical methods [18,19]. Beyond that, electrochemical sensors have been of great interest for future development. Electrochemical sensors do provide fast readouts, simple design, are cheap and easy to carry, making them a very exciting technology. In addition, these sensors can be miniaturized and made convenient to allow real-time detection of target analytes by non-specialist persons [20,21].

In recent times, electrochemical sensors derived from screen-printed carbon electrodes (SPCEs) have received great interest in research. This is due to the incredible benefits SPCEs have over traditional sensors, such as miniature, field-deployable size, portability, ease of production and cost. The SPCE surface is small and can therefore have a high active surface, making it very useful for analysing samples with low pH levels. In this way, screen-printed carbon electrodes are highly advantageous for their ease of use, reproducibility and reliability as well as excellent analytical behaviour demonstrating high sensitivity, greater selectivity and reproducible performance [22,23].

Yet, it is still a challenge to measure the EP and AC simultaneously. The oxidation peak of these two compounds is not well separated in most electrodes, posing a challenge to accurate measurement. Over the last few years, significant advancements in nanotechnology and novel manufacturing processes of SPCEs have substantially enhanced their electrochemical performance [24].

Nano-structured materials offer an extremely high surface-to-volume ratio; this property makes them great candidates for sensing objects. As a consequence, different nanoarchitectures have demonstrated better performance in applications such as sensor technology and optoelectronic devices. The properties of nanomaterials can be fine-tuned for a specific application by adjusting their dimensions or geometric shape [25,26]. Electroanalytical properties are determined with great significance by the surface characteristics of the material, since they directly affect key factors such as selectivity and sensitivity, stability or response speed. In general, nanomaterial-modified electrodes are widely used in analytical applications due to their higher selectivity and sensitivity [27,28].

Over the years, metal oxide nanoparticles (NPs) have found significant use in various scientific fields. This is largely based on their precautions for adjusting its physicochemical characteristics with exact control of particle size, structural morphology and crystalline face [29–31]. In particular, nickel oxide (NiO) nanostructures have continued to attract scientific attention during the past two decades. These factors can be attributed to high theoretical capacity, low cost, non-toxicity, high stability and abundance. Of particular note, NiO's remarkably high surface area makes it one of the best-known materials with catalytic activity. Additionally, NiO nanostructures are p-type semiconductors that have been useful in various physicochemical applications due to their wide band gap (3.6 to 4.0 eV). Which are pandas for solar cells 32P21, fuel cell electrodes 33P22, adsorbent materials 34P23, magnetic agents and gas & electrochemical sensors [32-34].

Herein, we fabricated NiO nanostructures via a simple and economical hydrothermal method. These nanostructures were subsequently used to fabricate a screen-printed carbon electrode, yielding a NiO/SPCE sensor. The NiO/SPCE sensor was then used as an electrocatalytic platform for the simultaneous determination of EP and AC. The NiO/SPCE sensor exhibited an impressive wide linear detection range from 0.01 to 400.0 μmol L-1 for EP with extraordinarily low limit of detection (LOD) of 0.005 μmol L^-1^ through differential pulse voltammetry (DPV). In addition, the NiO/SPCE sensor was successfully applied to detect EP and AC. The sensor is also successful at identifying EP and AC within relevant sample matrices.

## Experimental

### Chemicals and instruments

A phosphate buffer solution (PBS) was prepared using ortho-phosphoric acid and adjusted to different pH values. The pH of the solution was fine-tuned by adding an appropriate amount of sodium hydroxide solution. All chemicals used were of analytical-grade purity. In this experiment, all aqueous solutions were fabricated utilizing ultrapure water generated by a Millipore Direct-Q 8UV system (Germany; Darmstadt).

All electrochemical experiments were conducted at ambient temperature using a Metrohm PGSTAT302N potentiostat (Netherlands), operated with GPES version 4.9. A commercial screen-printed carbon electrode (DropSens model DRP-110, Spain) was used, featuring an integrated three-electrode configuration comprising a carbon working electrode, a silver pseudo-reference electrode, and a carbon counter electrode. The pH of all solutions was determined using a digital pH meter (Metrohm model 713; Switzerland).

### Synthesis of NiO nanostructures

At first, NiCl_2_·6H_2_O (0.0760 g) was dispersed into 80 mL of distilled water by magnetic stirring; then 0.0582 g cetyltrimethylammonium bromide (CTAB) was added to the above solution, followed by stirring until it completely dissolved. A few drops of ammonium hydroxide solution were added dropwise to the above solution, and the mixture was continuously stirred at room temperature for 2 hours. Subsequently, the prepared solution was transferred into a Teflon-lined stainless-steel autoclave and heated under hydrothermal conditions at 160 °C for 24 h. The precipitated solid products were centrifuged and washed several times with distilled water and ethanol to remove impurities. Finally, the product was dried in an oven at 70 °C for 15 h and then calcined at 300 °C for 3 h to obtain NiO nanostructures.

### NiO nanostructures modified-screen-printed carbon electrode

The SPCE was modified with NiO nanostructures. A stock suspension of NiO nanostructures was prepared by adding 1 mg of the material to the 1 mL of aqueous solution. It was sonicated for a period of 1 hour. A volume of 3 μL of the aqueous suspension of NiO nanostructures was cast onto the surface of the carbon working electrode. Finally, the solvents evaporated at room temperature, yielding the modified electrode (NiO/SPCE). To estimate the electroactive surface area (*A*) of bare SPCE and modified-SPCE, CV studies were performed at various scan rates in a 0.1 mol L^-1^ KCl solution containing 5.0 mmol L^-1^ [Fe(CN)_6_]^3-/4-^. Based on these studies and the Randles-Ševčik equation, the values of *A* were determined as 0.0321 cm^2^ for bare SPE and 0.086 cm^2^ for modified-SPCE.

## Results and discussion

### Characterization of NiO nanostructures

**Figure 1.**
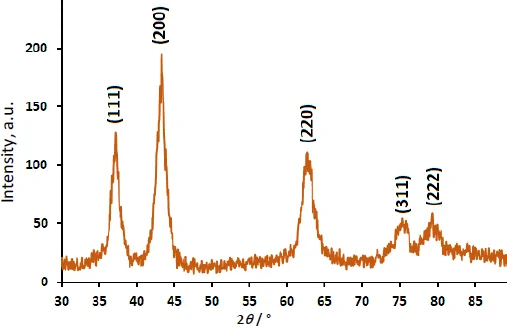
XRD pattern of NiO nanostructures

The XRD pattern of NiO nanostructures is exhibited in Figure 1. The observed peaks in the XRD pattern of NiO nanostructures located at 2*θ* = 37.1, 43.3, 62.6, 75.5 and 79.3° can be indexed as (111), (200), (220), (311) and (222) to a cubic structure of NiO (JCPDs No. 01-075-0197).

### Electrochemical behaviour of epinephrine at the NiO/screen-printed carbon electrode

The impact of pH variation within the buffer solution on the peak current response during EP detection was examined. This study employed a NiO/SPCE over a pH range of 2.0 to 9.0, using DPV for the measurements. Based on the findings, the anodic peak current (*I*_pa_) increased with elevated pH, reaching its maximum at pH 7.0, beyond which it declined with further increases in alkalinity. Accordingly, all subsequent experimental procedures were conducted with EP solutions formulated in 0.1 mol L^-1^ PBS at pH 7.0.

**Figure 2.**
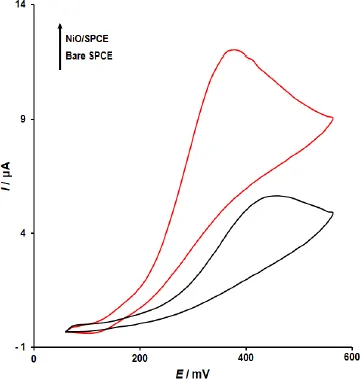
CV curves of 100.0 μmol L^-1^ EP in 0.1 mol L^-1^ PBS (pH 7.0) by bare SPCE and NiO/SPCE at scan rates of 50 mV s^-1^

An unmodified SPCE (a) and NiO/SPCE (b) were utilized for the electrochemical evaluation of EP. CV measurements were conducted with a sweep rate of 50 mV s-1 in 0.1 mol L-1 PBS at pH7.0. The Cu concentration of 100.0 μmol L−1 EP in 0.1 mol L−1 PBS at pH 7.0 is presented in Figure 2, which compares the responses of the unmodified electrode and NiO/SPCE, and shows the CV data for this comparison along with some results from a modified electrode control experiment. A noticeable oxidation peak at 450 mV was found for the unmodified SPCE electrode with EP concentration of 100.0 μmol L^−1^ In sharp contrast, the NiO/SPCE exhibited only a well-defined oxidation peak at 370 mV. The respective *I*_pa_ measured were 5.7 μA and 12.0 μA for unmodified SPCE and NiO/SPCE, respectively. This notable increase in *I*_pa_ at the NiO/SPCE is probably attributable to the larger electroactive surface area afforded by the presence of nanostructured NiO.

### Influence of scan rate

**Figure 3.**
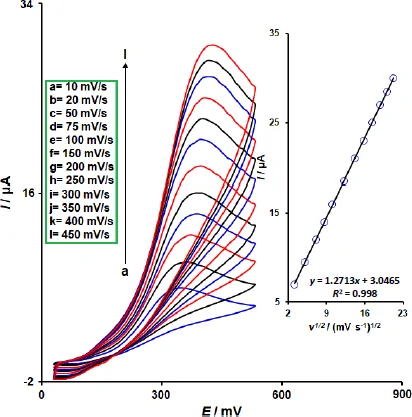
CV curves of 100.0 μmol L^-1^ EP in 0.1 mol L^-1^ PBS (pH 7.0) by NiO/SPCE at diverse scan rates from 10 mV s^-1^ to 400 mV s^-1^.; inset: the linear relationship between *I*_pa_ and the *v*^-1/2^

To examine the effect of scan rate, cyclic voltammograms of the NiO/SPCE were recorded at scan rates (*v*) ranging from 10 to 450 mV s^-1^ (Figure 3).

A positive shift in the peak potential was observed with increasing scan rate. The anodic peak current (*I*_pa_) linearly increased with an increase in the square root of scan rate (*v*^-1/2^) and followed the following calibration equation: *I*_pa_ = 1.2713*v*^1/2^ + 3.0465 with *R*^2^ = 0.9998 (Inset Figure 3). In conclusion, the linear relationship between *I*_pa_ and v^-1/2^ indicates that the electrochemical reaction is governed by a diffusion-controlled electron-transfer process.

### Chronoamperometric studies

**Figure 4.**
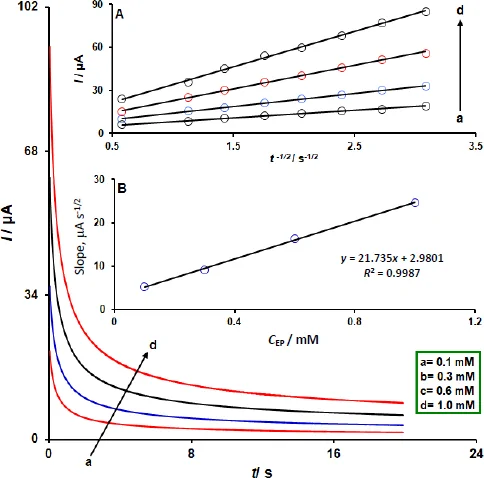
Chronoamperograms received at NiO/SPCE in PBS (pH 7.0; 0.1 mol L^-1^) for diverse concentrations of EP from 0.1 to 1.0 mmol L^-1^; insets: A) plots of *I*_pa_ against *t*^−1/2^; B) graph of the linear plot slopes against EP concentration



The chronoamperometric analysis was performed by holding the potential of the NiO/SPCE sensor at 420 mV for a solution containing 0.1-1.0 mmol L^-1^ EP in 0.1 mol L^-1^ PBS at pH 7.0, as illustrated in Figure 4. The current responses (*I*_pa_) for the diffusion-controlled electrochemical oxidation of the electroactive species (EP) were analysed using Cottrell's equation.

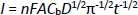

(1)


As shown in this Equation, the parameter *D* / cm^2^ s^-1^ represents the diffusion coefficient of the target analyte, while *C*_b_ / mol mL^-1^ denotes its bulk concentration. By plotting *I*_pa_ against the inverse square root of time (*t*⁻^1/2^), a linear relationship was derived from the chronoamperometric data for various concentrations of EP, as shown in Figure 4A. Subsequently, the slopes of these linear plots were graphed as a function of EP concentration, as presented in Figure 4B. Consequently, the diffusion coefficient for EP was calculated to be 4.0×10^-5^ cm^2^ s^-1^.

### Quantitative measurements of epinephrine at NiO/screen-printed carbon electrode sensor using differential pulse voltammetry

**Figure 5.**
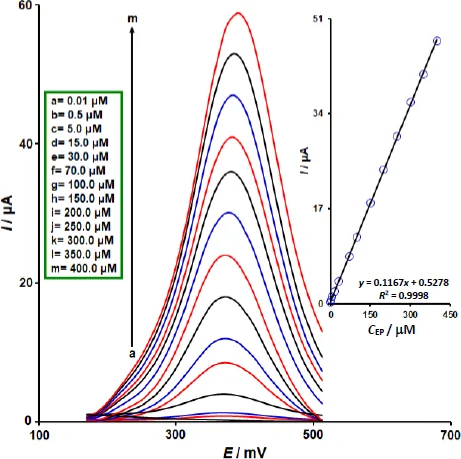
DPV responses obtained at the NiO/SPCE in PBS (pH 7.0; 0.1 mol L^-1^) containing various concentrations of EP (0.01 to 400.0 μmol L^-1^); inset: plot of the *I*_pa_ *vs*. EP concentrations

A calibration curve for EP in PBS (pH 7.0; 0.1 mol L^-1^) was constructed using DPV measurements with the NiO/SPCE under optimized conditions. Figure 5 displays representative DPVs recorded at various EP concentrations. The relationship between *I*_pa_ and EP concentration demonstrated that the *I*_pa_ scaled linearly with increasing EP concentration. The DPV results also indicated a linear response for EP over the range of 0.01 to 400.0 μmol L^-1^, as represented by the calibration equation *I*_pa_ = 0.1167*C*_EP_ + 0.5278. The LOD was determined to be 0.005 μmol L^-1^.

### The simultaneous determination of epinephrine and acetaminophen at NiO/screen-printed carbon electrode sensor

**Figure 6.**
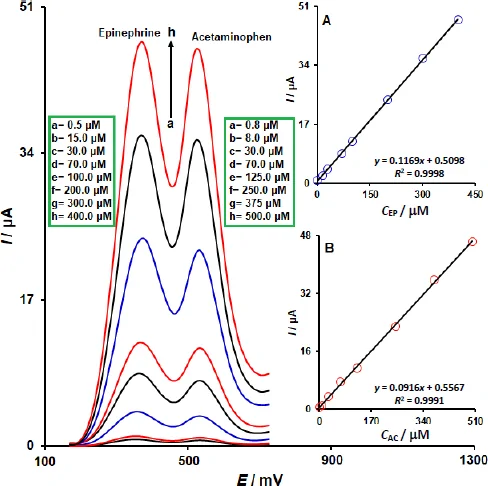
DPVs accepted NiO/SPCE in PBS (pH 7.0; 0.1 mol L^-1^) containing diverse concentrations of EP (from 0.5 μmol L^-1^ to 400.0 μmol L^-1^) and AC (from 0.8 μmol L^-1^ to 500.0 μmol L^-1^). Insets: A) plot of the *I*_pa_ versus. EP concentrations. B) Plot of the *I*_pa_ versus. AC concentrations

Simultaneous detection of EP and AC was performed using DPV in PBS (pH 7.0, 0.1 mol L^-1^) with the NiO/SPCE sensor. As illustrated in Figure 6, two clearly separated and well-defined oxidation peaks corresponding to EP and AC were observed. The peak currents for both analytes increased linearly with concentration, indicating the absence of significant interferences between them. As shown in Figure 6, two distinct peaks were observed at about 360 mV and 545 mV in the DPV signals for EP and AC, respectively. A peak separation of 185 mV between EP and AC enables their simultaneous detection via DPV. The sensitivity of the NiO/SPCE sensor for the oxidation of EP in a mixture containing both compounds was determined to be 0.1169 μA L μmol^-1^ (Inset A). This value is very close to the sensitivity measured for EP alone (0.1167 μA L μmol^-1^), indicating minimal interference from AC. This result confirms the feasibility of simultaneously quantifying EP and AC using the NiO/SPCE sensor.

### Application of the NiO/screen-printed carbon electrode platform for epinephrine and acetaminophen analysis in real water sample

The practical utility of the NiO/SPCE sensor for detecting trace amounts of EP and AC in real samples, specifically, urine and pharmaceutical products, was confirmed through recovery studies. As presented in Table 1, excellent recovery values, ranging from 96.7 to 103.4 %, were achieved for both urine samples and pharmaceutical formulations. These analytical recovery results demonstrate that the NiO/SPCE sensor is suitable for accurately measuring EP and AC concentrations in urine samples and pharmaceutical products.

**Table 1.** Voltammetric determination of EP and AC in real specimens at NiO/SPCE (*n* = 5)

Sample	Added concentration, μmol L^-1^		Found concentration, μmol L^-1^		Recovery, %		RSD, %	
Human urine	EP	AC	EP	AC	EP	AC	EP	AC
	0	0	-	-	-	-	-	-
	5.0	5.5	4.9	5.6	98.0	101.8	1.9	3.3
	7.0	7.5	7.1	7.3	101.4	97.3	3.1	2.1
	9.0	9.5	8.8	9.8	97.8	103.2	2.6	2.9
	11.0	11.5	11.3	11.4	102.7	99.1	2.2	3.2
Epinephrine Injection	0	0	1.9	-	-	-	3.0	-
	3.0	4.0	4.8	4.1	98.0	102.5	2.7	3.3
	4.0	6.0	6.1	5.8	103.4	96.7	2.2	2.6
	5.0	8.0	7.0	7.8	101.4	97.5	3.5	2.1
	6.0	10.0	7.8	10.1	98.7	101.0	1.8	2.4

## Conclusions

In conclusion, a simple and sensitive electrochemical sensor based on a SPCE with NiO nanostructures was successfully created. NiO nanostructures provide large surfaces, abundant active sites, and promote rapid electron transfer, resulting in excellent electrocatalytic activity for the oxidation of EP. Consequently, the EP sensor's performance evaluation demonstrated a LOD of 0.005 μmol L^-1^, a broad linear dynamic range from 0.01 to 400.0 μmol L^-1^, and high sensitivity. Well-defined, distinct oxidation peaks for EP and AC were obtained, enabling simultaneous detection and quantification of both substances using the NiO/SPCE platform. Finally, recovery rates between 96.7 and 103.4 % across all real-world samples validated the method's effectiveness for quantifying EP and AC in both urine and pharmaceutical products.
